# Toxic Effects of Sulfur Dioxide: A Review

**DOI:** 10.3390/toxics14010100

**Published:** 2026-01-21

**Authors:** Connor B. Stauffer, John Tat

**Affiliations:** Department of Medicine, University of California, San Diego, CA 92093, USA; cstauffer@ucsd.edu

**Keywords:** sulfur dioxide, air pollution, inhalation exposure, respiratory toxicity, pulmonary disease, extrapulmonary disorders, oxidative stress, gasotransmitter, environmental monitoring, toxicology

## Abstract

Sulfur dioxide (SO_2_) is a colorless, pungent gas that is a significant contributor to air pollution, with well-documented environmental and health impacts. It is emitted both naturally (e.g., in volcanic activities) and anthropogenically (e.g., fossil fuel combustion, sulfuric acid production, papermaking, and wine preservation). Inhalation represents the primary route of human exposure, particularly in urban and industrial settings. Acute SO_2_ exposure can lead to airway irritation, laryngospasm, bronchoconstriction, pulmonary edema, and death in severe cases. Chronic exposure, even at low concentrations, can contribute to the development of pulmonary and extrapulmonary diseases. Despite its classification as a hazardous air pollutant, a comprehensive understanding of dose-response relationships, exposure thresholds, and mechanisms of toxicity for SO_2_ remains limited. This review synthesizes current knowledge on environmental sources, exposure routes, mechanisms of toxicity, and health impacts of SO_2_, highlighting findings from epidemiological, toxicological, and mechanistic studies. We also discuss gaps in knowledge regarding SO_2_, approaches to monitor and assess SO_2_ exposure in ambient environments, the emerging role of SO_2_ as a gasotransmitter, and areas where further research is needed to better understand health risks and support evidence-based public health decision-making.

## 1. Introduction

Sulfur dioxide (SO_2_) is a colorless, toxic gas with a sharp, pungent odor detectable at concentrations as low as 0.1 parts per million (ppm) [1]. Despite regulatory efforts in many parts of the world, SO_2_ remains a significant industrial and urban pollutant. It is classified by the United States Environmental Protection Agency (EPA) as one of “six criteria air pollutants” alongside carbon monoxide, lead, nitrogen dioxide, ozone, and particulate matter [2]. The EPA has established the National Ambient Air Quality Standards (NAAQS) for each of these pollutants, and, therefore, SO_2_ levels are strictly monitored and regulated by the EPA due to their harmful effects on public health and the environment [3]. Indeed, both short- and long-term SO_2_ exposures have been linked to a wide range of adverse health effects, including respiratory symptoms (e.g., wheezing, coughing, and shortness of breath), cardiovascular diseases, neurological disorders, and metabolic syndromes, as well as an increased risk of hospital admission and mortality.

The molecular basis of SO_2_ toxicity is multifaceted. As described in this review, once dissolved in the aqueous environment of the airway, SO_2_ forms acid species that dissociate and lower the pH of the airway surface liquid (ASL). Acid directly injures cells, activates chemoreceptors, induces inflammation, and drives oxidative stress that can result in airway injury. We will also review the reported non-respiratory effects of SO_2_ exposure and their potential underlying mechanisms. Finally, we review emerging evidence suggesting that low, endogenous concentrations of SO_2_ may serve several physiological signaling functions, underscoring the dual toxic and regulatory nature of this molecule.

Beyond its biological role, SO_2_ is easy to make by mixing sodium sulfite (Na_2_SO_3_) with sulfuric acid (H_2_SO_4_), both of which are commercially available. Because SO_2_ is easily manufactured, widely used in industrial processes, and poses potential risks if misused, it is designated by certain governments, such as the United States Department of Homeland Security, as a Chemical of Interest (COI) under the Chemical Facility Anti-Terrorism Standards (CFATS), reflecting its relevance to both environmental health and national security [4].

In this review, we synthesize findings from epidemiological research, case reports, and mechanistic studies to advance understanding of SO_2_ toxicity and its public health implications.

## 2. Materials and Methods

Since this review is a narrative synthesis rather than a systematic review and meta-analysis, the literature was identified through exploratory searches of PubMed, Perplexity.ai Pro, and Google without predefined search strategies, time frames, or formal inclusion/exclusion criteria. Articles were selected based on their relevance to the topic and their contribution to conceptual understanding. The sole restriction was that only English-language articles were considered.

**Figure 1:**
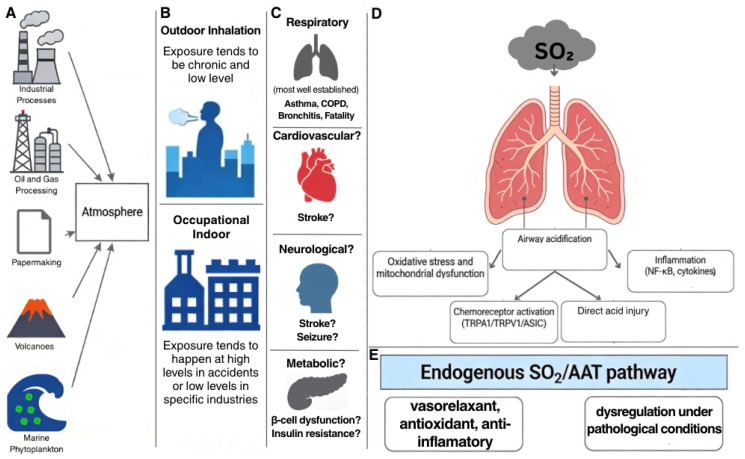
Schematic overview of SO_2_ sources, exposure pathways, and health effects. (**A**) Some of the sources of SO_2_. (**B**) Exposure scenarios spanning ambient urban levels to acute occupational/industrial incidents. (**C**) Health effects of SO_2_ based on various studies. Respiratory symptoms are more well-defined. Cardiovascular, neurological, and metabolic effects have been reported but are less concrete. (**D**) Proposed mechanisms of SO_2_ toxicity. (**E**) Roles of the endogenous SO_2_/AAT pathway and the proposition that it may be dysregulated under pathological conditions.

Regarding schematics, artificial intelligence (AI) via Perplexity.ai Pro was used exclusively for brainstorming concepts, and Google Gemini (Nano Banana Pro) was used for generating individual images representing key elements, including industrial processes, natural sources (e.g., volcano and marine plankton), and target organs (e.g., brain, heart, and lung), ensuring that copyright issues were avoided. Images were generated with custom prompts tailored to depict each concept. These AI-generated images were then assembled, annotated, and finalized into cohesive schematics (i.e., graphical abstract and Figure 1) using a digital design software (Canva, https://www.canva.com).

## 3. Results

### 3.1. Sources of SO_2_

Globally, approximately 55 million tons of SO_2_ are released each year from both natural and anthropogenic sources (Figure 1A) [5,6]. Large amounts of SO_2_ are released in volcanic activity through eruptions and continuous venting from fumaroles [7]. Other geothermal sources, such as hot springs and geysers, also release sulfur gases, including SO_2_ and hydrogen sulfide (H_2_S) [8]. The oxidation of H_2_S to SO_2_ is a well-known atmospheric process.

Marine phytoplankton contribute indirectly to atmospheric SO_2_ by producing dimethyl sulfide (DMS) gas [9], which escapes into the atmosphere and oxidizes to SO_2_ and sulfate (SO_4_^2−^) aerosols. Natural wildfires in forests and grasslands emit SO_2_ as organic matter burns [10]. In oxygen-deprived wetlands such as swamps and marshes, sulfate-reducing microorganisms decompose organic matter and generate H_2_S as a key product of anaerobic metabolism, contributing to the sulfur cycle that sustains rapid sulfur turnover in these ecosystems [11]. Again, H_2_S can be oxidized to SO_2_ in the atmosphere.

While natural emissions from volcanoes, marine processes, and wildfires are substantial, human activities have been the primary driver of SO_2_ pollution since the onset of the Industrial Revolution. SO_2_ is primarily produced through industrial processes involving the combustion of sulfur-containing fossil fuels, and to a lesser extent, sulfuric acid manufacturing [12], wine preservation [13], and papermaking [14]. Countries that generate the greatest amount of SO_2_ are India (due to coal-based electricity generation) and Russia, Iran, and Saudi Arabia (due to oil and gas processing) [15].

### 3.2. Atmospheric Fate of SO_2_ and Environmental Impact

Once released into the atmosphere, SO_2_ undergoes oxidation, often by hydroxyl radicals (·OH) in the presence of water vapor, to form sulfuric acid [16]. Sulfuric acid then condenses onto existing atmospheric particles (e.g., sea salt, dust, and black carbon) or nucleates new fine-mode sulfate (SO_4_^2−^) aerosols. These aerosols have dual environmental effects. (A) Climate cooling: SO_4_^2−^ aerosols can scatter incoming solar radiation and increase the reflectivity of clouds by acting as cloud condensation nuclei. This phenomenon results in a net cooling effect on the Earth’s surface, masking some of the warming caused by greenhouse gases. This effect has been observed in the aftermath of large volcanic eruptions, such as Mount Pinatubo in 1991, which temporarily lowered global temperatures by about 0.5 °C [17]. (B) Acid rain: SO_4_^2−^ aerosols can return to Earth as a component of acid rain, which significantly lowers soil and water pH. The acidification can damage terrestrial and aquatic ecosystems, as well as agricultural productivity. Acid rain also accelerates the corrosion of buildings and monuments, especially those made from limestone or marble.

SO_4_^2−^ aerosols constitute a major component of fine particulate matter (e.g., PM_2.5_). Usually ≤2.5 microns in size, these particles penetrate deeply into alveoli and, as a major component of urban smog, are strongly linked to respiratory exacerbations, cardiovascular stress, and excess mortality [18,19,20]. This atmospheric transformation creates a dual toxic exposure profile: acute SO_2_-induced airway inflammation coupled with chronic PM_2.5_-driven systemic oxidative stress. The gas-to-aerosol transition may explain the lag between peak SO_2_ emissions and respiratory-related hospital admissions described later in the review, with bronchoconstriction occurring within hours following SO_2_ exposure, but neutrophil recruitment and adaptive immunity unfolding over days [21,22]. This temporal mismatch is critical for interpreting epidemiological time-series and acute-exposure animal models, which often miss the full toxic cascade.

### 3.3. SO_2_ Exposure Assessment Methods

Accurate measurement of ambient SO_2_ concentrations is essential for characterizing human exposure, supporting dose-response evaluations, and informing public health interventions. Several monitoring approaches are available, but each has trade-offs in sensitivity, specificity, portability, and cost.

Cavity ring-down spectroscopy (CRDS) provides sub-parts per billion (sub-ppb) sensitivity for research needing high-resolution data (e.g., low-level chronic risk models) [23]. However, high cost, technical complexity (e.g., temperature and vibration sensitivity) [24], and limited portability restrict routine epidemiological surveillance. Portable electrochemical sensors are widely used for workplace safety and personal monitoring because of their size, accuracy, affordability, and robustness [25]. However, humidity, temperature, and electrode degradation can cause drift [26,27], raising concerns for chronic, low-level assessments where underestimation could bias epidemiological risk estimates. Continuous ultraviolet fluorescence analyzers (EN 14212 standard [28]) are the primary instruments used in ambient air monitoring, working by exciting SO_2_ with ultraviolet light, causing SO_2_ to fluoresce [29]. However, high costs and fixed-site deployment limit spatial coverage, potentially missing hotspots or micro-environmental exposures that drive acute health events such as asthma flares. Automated titration systems, high-performance liquid chromatography, and ion chromatography can quantify dissolved sulfite and sulfate species in industrial or food matrices [30,31,32,33,34]. However, these instruments are costly, cumbersome, and technically complex, thus requiring skilled operators and exhibiting slow analytical rates, which makes them impractical for field/rapid use. Also, because they do not measure airborne SO_2_, this limits their relevance to inhalation health risks. Colorimetric assays (e.g., the sulfite test strip and the West-Gaeke method) and manual titration methods (e.g., the Rankine and Ripper methods) provide low-cost, user-friendly alternatives for sulfite and sulfate determination [32]. However, they have lower sensitivity and specificity and greater read variability than instrumental methods. Interferences, such as from lighting and matrices, further compromise accuracy. Thus, they may be unreliable for low-level detection critical to public health protection.

Altogether, while numerous methods for detecting SO_2_ and its derivatized species exist, none optimally balances sensitivity, specificity, portability, cost, and real-time capability for comprehensive exposure assessment, especially in the multi-pollutant urban context. Given the health risks of even low-level SO_2_ exposures, critical gaps persist in developing affordable, sensitive, and portable detectors to refine epidemiological dose-responses. Such improvement in SO_2_ detection could bridge toxicology to policy.

### 3.4. Respiratory Illnesses and Fatalities Due to Short-Term SO_2_ Exposure

SO_2_ exposure occurs via three main routes: dermal contact, ocular contact, and inhalation. Inhalation is the most common and serious exposure route and can lead to airway irritation, laryngospasm, bronchoconstriction, pulmonary edema, and death (Figure 1C) [35]. Humans can tolerate ~100 ppm of SO_2_ for 30 min before succumbing to the gas [36].

Epidemiological studies indicate SO_2_ exposure exerts harmful effects on respiratory health even at low-to-moderate ambient concentrations (Table S1). For example, Samoli et al. found that a 10 µg/m^3^ increase in ambient SO_2_ levels in Athens, Greece, was associated with a 5.98% (95% confidence interval [CI]: 0.88–11.33%) increase in pediatric asthma hospital admissions [37]. Meanwhile, in their systematic review and meta-analysis of 15 articles, Zhou et al. reported that the relative risk (RR) of chronic obstructive pulmonary disease (COPD) was 1.26 (95% CI: 0.94–1.70) per 10-μg/m^3^ increase in ambient SO_2_ levels [38].

In a time-series analysis of 12 European cities, Katsouyanni et al. reported that a 50 µg/m^3^ increase in SO_2_ levels partially contributed to a 3% (95% CI: 2–4%) increase in daily mortality [39]. As part of the China Air Pollution and Health Effects Study (CAPES), Chen et al. analyzed data from 17 Chinese cities and showed that an increase of 10 μg/m^3^ (measured as a two-day moving average) in SO_2_ levels resulted in a 1.25% (95% posterior interval [PI], 0.78–1.73) increase in respiratory mortality [40]. A nationwide, time-series study across 48 Chinese cities covering approximately 403 million people by Li et al. found that each 10 µg/m^3^ increase in SO_2_ levels (measured as a four-day moving average) was linked with a 0.83% (95% CI: 0.13–1.53%) rise in years of life lost (YLL) due to COPD and a 0.78% (95% CI: 0.16–1.41) rise in related mortality [41]. Finally, a systematic review and meta-analysis by Orenello et al. showed a positive correlation between a 10 µg/m^3^ increase in SO_2_ levels and all-cause and respiratory mortality [42]. These lines of evidence establish that even modest, short-term SO_2_ exposures contribute to pulmonary morbidity and mortality, underscoring the importance of stringent air quality standards. However, while the data indicate a link between short-term SO_2_ exposure and pulmonary disorders and lung-related deaths, these estimates often arise from highly polluted urban environments with substantial co-pollutants (e.g., nitrogen oxides, ozone, and particulate matter), making it difficult to identify SO_2_-specific effects.

Clinical case reports have documented that single, high-dose SO_2_ exposures can cause lasting and, in some instances, irreversible lung damage. Woodford et al. reported that a healthy, non-smoking young man experienced acute pulmonary edema after a brief but intense exposure to an unknown but likely high concentration of SO_2_ [43]. Although his symptoms initially resolved, the patient later developed severe, irreversible obstructive lung disease. Imaging and lung function tests showed findings consistent with bronchiolitis obliterans and permanent scarring of the small airways, pointing to toxic inhalation as the most plausible etiology, although precise exposure levels and co-exposures could not be fully characterized. Rabinovitch et al. reported that two non-smoking miners developed severe airway obstruction following acute exposure to high concentrations of SO_2_, measured above 40 ppm, released during a mine explosion [44]. Within three weeks, both miners showed marked hypoxemia, impaired exercise tolerance, ventilation-perfusion mismatch, and active lung inflammation. While imaging and lung function tests revealed gradual improvement over the first year, neither patient’s pulmonary function returned to baseline, and their condition stabilized without further recovery over the following year. These case reports, although limited by small sample size and incomplete exposure characterization, reinforce that acute, high-dose SO_2_ inhalation can produce long-lasting airway injury in otherwise healthy individuals.

Fatalities have also occurred after one-time, high-dose SO_2_ exposure. Huber and Loving reported that a woman died after inhaling an estimated 150 ppm of SO_2_ from a de-rusting agent; postmortem findings of “empty” airways with mild mucus plugging were consistent with sudden asthmatic death [45]. Gorell reported that one of two workers died after SO_2_ exposure while cleaning a broiler in a smelting plant [46]. Charan et al. reported that two workers died after the valve on a pipe containing SO_2_ was accidentally opened [47]; one of the deceased individuals exhibited hemorrhagic alveolar edema. Although these fatal cases rarely provide complete information on co-exposures or dose-response, they illustrate that under certain occupational or accidental circumstances, SO_2_ concentrations can rapidly reach life-threatening concentrations. Taken together, high-dose SO_2_ exposure produces clear and often severe toxicological effects. Short-term ambient exposures, however, have more modest effects in the general population, whereas susceptible individuals may exhibit heightened responses due to factors such as preexisting airway inflammation (e.g., asthma) [48].

### 3.5. Respiratory Illnesses Due to Chronic SO_2_ Exposure

Chronic exposure to SO_2_ is linked to a broad spectrum of respiratory illnesses, including chronic bronchitis, obstructive lung disease, and lung cancer, especially among occupationally exposed populations (Table S2). After adjusting for co-exposures, Lee et al. found an elevated lung cancer risk (RR: 1.49, 95% CI: 1.14–1.96) in a multi-national cohort of over 40,000 SO_2_-exposed pulp and paper workers [49]. A 30-year cohort study of 3060 Swedish pulp mill workers, reported by Andersson et al., concluded that repeated SO_2_ “gassing” episodes more than doubled the incidence of chronic bronchitis (hazard ratio [HR]: 2.1, 95% CI: 1.4–3.1); even never-smokers among the studied individuals experienced an elevated risk of developing bronchitis if they had frequent exposures (HR: 8.7, 95% CI: 3.54–22) [50]. The combined epidemiological findings underscore the significant respiratory health burden posed by chronic SO_2_ exposure, suggesting that stringent regulatory measures and workplace protections may be warranted for workers who are repeatedly exposed.

Among pediatric patients, Herbarth et al. found that lifetime SO_2_ exposure strongly increased bronchitis prevalence in a cohort of approximately 3800 East German children (odds ratio [OR]: 3.51, 95% CI: 2.56–4.82), indicating the cumulative impact of chronic exposure during childhood [51]. A case-control study by Lin et al. found that short-term exposure to ambient SO_2_ levels significantly increased hospitalizations for childhood asthma in Bronx County, New York City, with odds ratios as high as 2.21 for a 3-day lag [52]. Altogether, chronic and short-term SO_2_ exposures have significant, but perhaps distinct, adverse respiratory impacts in children. Lifetime exposure seems to substantially increase the risk of chronic bronchitis, while brief spikes in ambient SO_2_ levels may acutely worsen asthma, leading to increased hospitalizations. However, both relationships derive from complex environmental contexts where SO_2_ co-occurred with other pollutants, complicating causal attribution. To address concerns regarding confounding in observational studies, mechanistic studies in animal models where isolated and repeated SO_2_ exposure is possible demonstrate that chronic inhalation damages the airway epithelium, triggers mucus hypersecretion, and promotes airway remodeling, providing biological plausibility for the epidemiologic associations [53,54]. These experimental findings, nonetheless, cannot delineate the dose-response relationships or establish causality attributable to SO_2_ alone, creating a gap in knowledge.

### 3.6. Extrapulmonary Effects of SO_2_ Exposure

While respiratory distress is the chief complaint among patients exposed to SO_2_, extrapulmonary effects have also been documented (Figure 1C) (Table S3). A case-crossover study in Vancouver, Canada, by Szyszkowicz et al. reported that short-term increases in ambient SO_2_ were associated with a 12% increase in the odds of emergency department (ED) visits for ischemic stroke per interquartile range (IQR) (1.9 ppb) increase in SO_2_ (OR: 1.12, 95% CI: 1.02–1.23; lag 3) [55]. Among women, short-term ambient SO_2_ exposure was also linked to an 18% increase in seizure-related ED visits per interquartile range increase (OR: 1.18, 95% CI: 1.05–1.32; lag 2) [55]. Liu et al. found that every 10.1 μg/m^3^ increase (IQR, lag 0–1 days) in daily average ambient SO_2_ across 14 Chinese cities increased the risk of ischemic stroke hospitalizations by 1.6% (95% CI: 1.0–2.3%), with no significant hemorrhagic stroke association [56]. Finally, Wu et al. showed a 0.7% increase in total stroke years of life lost per 10 μg/m^3^ increase in daily ambient SO_2_ (95% CI: 0.27–1.13%), especially in southern China and lower-education groups [57]. However, the extrapulmonary effects of SO_2_ exposure remain mechanistically uncertain and epidemiologically weak. Stroke and seizure studies were conducted in multi-pollutant urban settings where SO_2_ coexists with nitrogen oxides, ozone, and particulate matter, pollutants that are also linked to vascular dysfunction and neuro-inflammation [58,59,60,61]. Moreover, given these shared exposures and the constraints of case-crossover and time-series designs (e.g., high pollutant collinearity, exposure misclassification, and limited ability to infer causality), isolating SO_2_’s independent effect or establishing causality is not possible. Thus, SO_2_ cannot be classified as an isolated neurovascular toxicant at this time.

A positive correlation exists between SO_2_ exposure and type 2 diabetes mellitus (T2DM) risks. A 12-year cohort study by Shan et al. concluded that each 10 µg/m^3^ increase in SO_2_ exposure was linked to a 29% increase in the hazard of developing diabetes (95% CI: 1.26–1.32) and a 13% increase in the hazard of mortality (95% CI: 1.04–1.23) [62]. A study by Wu et al. found that short-term exposures per 10 µg/m^3^ increase in SO_2_ levels were linked to a 3.84% increase in daily diabetes mortality (95% CI: 1.48–6.19); the impact was stronger in women and adults aged 65 and above [63]. Finally, Li et al. reported that long-term exposure to SO_2_ was associated with a modest but statistically significant increase in the risk of developing T2DM. Specifically, each interquartile range increase in SO_2_ (1.77 ppb) corresponded to a hazard ratio of 1.011 (95% CI: 1.007–1.015) [64]. The metabolic effect estimates are generally small and arise in contexts where SO_2_ occurs with other pollutants, effect sizes are modest (HR: 1.01–1.29 per IQR increase; synthesized from Li et al., Shan et al., and Wu et al. [62,63,64]), confidence intervals are narrow but overlap in some studies, and no dose-response is apparent across studies differing in exposure levels and populations. This pattern is consistent with residual confounding (SO_2_ as a marker of overall air pollution burden) rather than direct SO_2_-mediated metabolic toxicity. Notably, no controlled animal inhalation studies evaluating SO_2_’s metabolic toxicity at ambient-relevant exposure levels have been published, leaving a gap between epidemiologic associations and mechanistic evidence. Current evidence, accordingly, does not support classifying SO_2_ as a distinct diabetogenic agent. These extrapulmonary associations should therefore be interpreted cautiously and indicate the need for targeted mechanistic investigations.

### 3.7. Mechanistic Underpinning of SO_2_ Toxicity

#### 3.7.1. Proposed Mechanisms for the Pulmonary Effects of SO_2_

*Acid generation*: Once dissolved in the aqueous environment of the airway, SO_2_ primarily forms sulfurous acid (H_2_SO_3_) and secondarily sulfuric acid through further oxidation. However, local formation of H_2_SO_4_ in the airway is considered minimal compared to H_2_SO_3_. H_2_SO_3_ rapidly dissociates at physiological pH to hydrogen (H^+^), bisulfite (HSO_3_^−^), and sulfite (SO_3_^2−^) ions, and oxidation of sulfite can yield sulfate [35]. H_2_SO_3_, and to a lesser extent, H_2_SO_4_, can directly irritate lung tissues, triggering bronchoconstriction, inflammation, and cellular damage [48]. Because this chemistry occurs in the airway surface liquid, the resulting local fall in ASL pH may be a key driver of epithelial injury (Figure 1D).

*Acid-induced cellular injury*: Experimental acidification of human bronchial epithelial (16HBE) cells with hydrochloric acid (HCl) to pH values below 5.0 reduced transepithelial electrical resistance and increased tight junction protein degradation, indicating barrier disruption [65]. Exposing BEAS-2B bronchial epithelial cells to HCl, pH 4.0, reduced cell viability and increased both lactate dehydrogenase release and apoptosis [66]. In vivo, instillation of HCl solutions or aspirated gastric contents (typically with pH values below 3.0 in bolus models) produces acute epithelial necrosis, neutrophil-rich airway inflammation, and impaired alveolar function [67,68,69]. In particular, Takeuchi et al. reported that H_2_SO_4_ mist inhalation in rats produced dose-dependent epithelial necrosis, mucosal clefts, alveolar hemorrhage/edema, and neutrophil-rich inflammation, pathological changes that are qualitatively similar to HCl-induced injury [70]. These studies support the general principle that acidic environments directly injure airway epithelial cells. Although these experiments used hydrochloric acid (with limited sulfuric acid data), it is reasonable to infer that protons from sulfurous and sulfuric acids would have similar injurious effects on airway cells. However, the near-complete reliance on HCl surrogates represents an evidence gap that limits mechanistic confidence and highlights the need for direct H_2_SO_3_/H_2_SO_4_ exposure studies in relevant airway models.

*Acid-induced chemoreceptor activation*: Beyond direct cellular injury, acid also activates transient receptor potential ion channels (TRPA1, TRPV1) and acid-sensing ion channels (ASICs) and can indirectly affect calcium (Ca^2+^) channels [71,72,73]. These receptors are abundant in airway sensory nerves and epithelial cells, and their activation initiates inflammatory signaling cascades, membrane depolarization, and enhanced airway reactivity. Acidosis, therefore, triggers cough and bronchospasm [74,75].

*Acid-induced inflammation*: SO_2_ and its acidic metabolites can upregulate expression of pro-inflammatory genes, such as those encoding for tumor necrotic factor (TNF)-α, interleukin (IL)-1β, cyclooxygenase-2 (COX-2), intercellular adhesion molecule 1 (ICAM-1), and MUC5AC (airway mucin), which contribute to asthma pathogenesis and airway hyper-responsiveness [76,77,78]. Animal studies report that co-exposure to SO_2_ and allergens increases Th2 cytokines and eosinophilic inflammation far beyond what is seen with allergens alone [79].

Neurogenic inflammation occurs when chemical irritants activate chemoreceptors (TRPA1, TRPV1, and ASICs) on sensory nerves, triggering the release of neuropeptides that contribute to airway inflammation [80]. Exposure to SO_2_ and sodium metabisulfite induces bronchoconstriction through activation of capsaicin-sensitive sensory nerves, illustrating the role of sensory nerve-mediated pathways in airway responses to environmental chemicals [81]. At the molecular level, inflammatory mediators, including bradykinin, prostaglandins, and histamine, have the potential to further sensitize these ion channels, contributing to persistent airway hyperactivity, cough, and mucus secretion over time [71].

*Reactive oxygen species (ROS) generation and downstream effects*: ASL acidification can promote reactive oxygen species production, similar to the oxidative damage observed following HCl aspiration in animal models, which damages the bronchoalveolar tree and lung parenchyma [82,83,84]. Hydrogen peroxide (H_2_O_2_), generated endogenously in cells under oxidative stress, can oxidize protein cysteine residues to sulfenic acid (R-SOH), altering protein structure and function [85]. Sulfite-induced inflammation recruits leukocytes in vitro and neutrophils in vivo, thereby increasing NADPH oxidase activity and driving O_2_^−^ generation [86,87]. Consequently, oxidative tissue injury is a key pathway underlying SO_2_ toxicity, as evidenced by increased lipid peroxidation and protein carbonylation, markers of oxidative damage [88,89,90]. Rodent studies have confirmed these oxidative effects, showing impaired glutathione redox balance and suppression of antioxidant enzymes such as superoxide dismutase (SOD), glutathione peroxidase (GSH-Px), and glucose-6-phosphate dehydrogenase (G6PD), alongside elevated ROS levels [91,92]. Together, these oxidative insults promote persistent tissue damage, airway remodeling, and chronic lung disease exacerbation. ROS can also covalently activate TRPA1 channels at cysteine residues, creating a feedback loop wherein oxidative stress sensitizes irritant receptors, further intensifying pulmonary inflammation and dysfunction [71].

#### 3.7.2. Potential Mechanisms for the Extrapulmonary Effects of SO_2_

Chronic SO_2_ exposure appears to cause neurological defects primarily through oxidative stress, vascular compromise, and neuroinflammation. In a rat model, SO_2_-induced oxidative stress activates pro-inflammatory signaling, particularly NF-κB-mediated upregulation of cyclooxygenase-2 (COX-2), leading to increased prostaglandin synthesis, neuronal injury, and apoptosis [93]. SO_2_ exposure also promoted vascular compromise through elevated expression of endothelin-1 (ET-1), inducible nitric oxide synthase (iNOS), COX-2, and intercellular adhesion molecule-1 (ICAM-1), mediators that intensify ischemic brain damage similar to stroke [94].

Epidemiological data suggest that acute ambient SO_2_ exposure increases the risk of ischemic stroke and seizures, particularly among females [55,56]. Although not experimentally confirmed, Szyszkowicz et al. suggested that SO_2_ may breach the blood-brain barrier, resulting in cerebrovascular events and abnormal neural activity [55]. More broadly speaking, air pollution containing SO_2_ is associated with structural brain alterations, persistent neuroinflammation, and imbalances in neurotransmitters such as dopamine, serotonin, and GABA, culminating in neurodegenerative processes, cognitive decline, and other neurological disorders [61,95].

Similarly, the harmful effects of SO_2_ exposure on the cardiovascular system may be related to mitochondrial dysfunction due to reduced membrane potential, ATP depletion, and cytochrome c oxidase inhibition, leading to impaired oxidative phosphorylation and cellular energy failure [96]. These changes are associated with the downregulation of key mitochondrial biogenesis regulators, including PGC-1α, a master coactivator of genes involved in mitochondrial energy metabolism [97]; NRF1, a transcription factor that regulates the expression of mitochondrial DNA transcription and replication genes [98]; and TFAM, a protein essential for mitochondrial DNA maintenance and transcription [99]. In a rat model, mitochondrial impairment contributes to increased oxidative stress, which triggers the upregulation of pro-inflammatory genes such as TNF-α, IL-1β, iNOS, and ICAM-1 [76]. Additionally, an increased ratio of pro-apoptotic Bax to anti-apoptotic Bcl-2 protein leads to enhanced apoptosis in cardiac cells, further exacerbating tissue injury [76]. Yet, because these findings are derived from supra-physiological exposures (mg/m^3^ doses as opposed to the environmental μg/m^3^ doses), translation to ambient human levels or multi-pollutant contexts is uncertain.

Finally, ambient SO_2_ exposure has been associated with increased T2DM risk (Table S3) [62,63,64], consistent with the concept that pollutant-induced oxidative stress can disrupt insulin signaling and β-cell function to impair glucose homeostasis. However, inhalation-based animal models specifically designed to assess diabetogenic outcomes of SO_2_ exposure are still lacking, representing a mechanistic gap between human associations and laboratory evidence.

### 3.8. Is It All Bad? Emerging Physiological Roles for SO_2_

While SO_2_ exposure has traditionally been viewed as exogenous and harmful, emerging evidence suggests that SO_2_ is also endogenously generated through the metabolism of sulfur-containing amino acids [100,101]. This production is widespread and occurs in multiple tissues, including the cardiovascular, respiratory, nervous, digestive, urinary, and immune systems, indicating that endogenous SO_2_ may exert biological effects across different organs rather than being confined to a single cell type or organ [100,102,103,104]. In this endogenous context, SO_2_ may function as a gasotransmitter akin to nitric oxide, carbon monoxide, or hydrogen sulfide, modulating oxidative balance and regulating physiological processes across systems.

There are two main biochemical pathways responsible for endogenous SO_2_ generation. One major route begins with L-cysteine, which is oxidized by cysteine dioxygenase (CDO) to form L-cysteinesulfinate; the latter is transaminated by aspartate aminotransferase (AAT1 and AAT2) to yield β-sulfinylpyruvate. This intermediate spontaneously decomposes to SO_2_ and pyruvate [100]. Another major pathway involves the mitochondrial oxidation of H_2_S [105,106]. In this process, H_2_S is oxidized by sulfide quinone oxidoreductase (SQR) to produce a persulfide intermediate, which is converted by mitochondrial persulfide dioxygenase (ETHE1) to sulfite. Under physiological conditions, sulfite exists in equilibrium with SO_2_. Together, these mechanisms help regulate endogenous SO_2_ levels in mammalian tissues.

#### 3.8.1. Proposed Mechanism of Endogenous SO_2_-Mediated Vascular Protection

At physiological concentrations, endogenous SO_2_ protects vascular functions, including vasodilation, inhibition of vascular smooth muscle proliferation, maintenance of vascular structure, and anti-inflammatory and antioxidant activities. This is demonstrated using AAT-targeted manipulation (e.g., overexpression/inhibition with aminooxyacetic acid) and SO_2_ donor sodium sulfite in cystathionine γ-lyase/hydrogen sulfide-deficient models (Figure 1E) [100,104,107]. Mechanistically, endogenous SO_2_ generated via AAT-mediated transamination of L-cysteine can increase intracellular cyclic adenosine monophosphate (cAMP) and activate protein kinase A (PKA) [108]. PKA phosphorylates c-Raf at the inhibitory Ser259 site, thereby blocking c-Raf kinase activity and preventing downstream activation of the extracellular signal-regulated kinase (ERK)/mitogen-activated protein kinase (MAPK) signaling cascade, which drives vascular smooth muscle cell (VSMC) proliferation [108] and may contribute to the protection against vascular remodeling seen in animal models of hypertension [107]. This molecular pathway may explain why animal models with reduced endogenous SO_2_ levels showed hypertension and vascular structural remodeling, and restoration to physiological SO_2_ concentrations attenuates such pathologies [100,107]. Conversely, elevated SO_2_ concentrations, as seen in chronic urban exposure or industrial accidents, shift its role toward toxicity, highlighting the delicate balance required for its biological function [100,103].

#### 3.8.2. Interaction Between Exogenous SO_2_ Toxicity and the Endogenous SO_2_-AAT Pathway: A Conceptual Model

The endogenous SO_2_/AAT pathway may exert protective effects in the vascular endothelium and smooth muscle; meanwhile, exogenous SO_2_ exposure may impair this protective pathway (Figure 1E). For instance, endogenous SO_2_ levels and AAT activity decrease markedly in oleic acid-induced acute lung injury rat models, consistent with the downregulation of the endogenous SO_2_/AAT pathway [109]. Oxidative stress can also impair Nrf2 signaling [110], and in SO_2_-related oxidative injury models, reduced AAT activity may reflect both transcriptional downregulation and direct oxidative modification of the catalytic cysteine residues on AAT [111]. This progressive loss of endogenous SO_2_ weakens the vascular endothelium and smooth muscle protective signaling that normally counteracts pathological effects [100,107]. In hypertension and other chronic exposure models, this transition from acute to chronic SO_2_ disease, where initial neutrophilic inflammation and acid injury progress to sustained vascular damage, may elevate the risks for stroke, as the endogenous SO_2_’s “brake” on vascular inflammation is lost amid the ongoing exogenous ROS-driven pathology [100,103,107].

Taken together, the dual roles of SO_2_, both as a toxicant and a regulatory molecule, highlight the complexity of its biological effects and emphasize the need for further research. Currently, the incomplete understanding of SO_2_’s molecular pathways remains a key barrier to developing targeted treatments for SO_2_ toxicity.

## 4. Conclusions

SO_2_ is an air pollutant released from a variety of human activities and natural sources (Figure 1A) with exposure scenarios spanning ambient urban levels to acute occupational/industrial incidents (Figure 1B). Our findings align with those of Khalaf et al. in two key aspects [112]. First, we found that acute and chronic exposure to SO_2_ poses significant health risks, notably through respiratory toxicity characterized by bronchoconstriction, pulmonary inflammation, and exacerbation of conditions such as asthma and chronic bronchitis, as well as lung-related fatalities (Figure 1C). Second, we found evidence implicating SO_2_ exposure in broad systemic effects, including increased risks of cardiovascular, neurological, and metabolic disorders (Figure 1C). Our review extends the discussion by integrating mechanistic insights, evaluating monitoring methodologies, exploring emerging evidence of endogenous physiological roles, and highlighting explicit knowledge gaps that warrant future investigation.

The mechanism of toxicity for SO_2_ seems to be multifaceted (Figure 1D,E), involving a complex interplay of acid-induced epithelial damage, airway chemoreceptor activation, and inflammation alongside the well-known oxidative injury pathways. Toxicity may involve less well-stablished mechanisms, including potential dysregulation of endogenous SO_2_/AAT signaling. Chronic SO_2_ exposure may compromise this endogenous protective system, observed as reduced SO_2_/AAT levels in oxidative lung injury models [109]. It is also linked to vascular remodeling when endogenous SO_2_ is deficient [100,107], via mechanisms such as Nrf2 impairment [109] and AAT oxidative modifications [112] that remain to be fully delineated.

Despite ongoing regulatory efforts, SO_2_ remains a critical public health concern due to its persistence in urban environments and potential for both short- and long-term adverse outcomes. While high-dose effects are better understood, uncertainties remain about (1) specific health risks from chronic, low-level exposures, (2) detangling SO_2_’s independent contribution in multi-pollutant mixtures, and (3) the role of endogenous SO_2_ pathways in health and toxicity. Thus, SO_2_-specific risk estimates should be interpreted cautiously to reflect the fact that air pollution is a mixture of compounds, one of which is SO_2_.

Beyond SO_2_’s dual role as an endogenous gasotransmitter versus an exogenous toxicant, emerging evidence suggests air pollution exposure, which can include SO_2_ as a co-pollutant, induces epigenetic remodeling via DNA methylation at antioxidant/immunoregulatory genes (e.g., FOXP3 and cytokines) [113,114] and histone acetylation (e.g., H3K9ac and H3K27ac) at inflammatory loci [115]. These persistent changes may explain elevated disease risk years after exposure. While beyond this review’s scope, such reports warrant mention and further investigation to expand our understanding of SO_2_ biology.

Continued research, especially using advanced models such as lung organ-on-a-chip, precision-cut lung slices, and human-relevant animal models, is needed to further elucidate molecular pathways, improve exposure monitoring, and develop stringent emission controls for mitigating SO_2_’s health burden and protecting vulnerable populations, e.g., children and factory workers. Moreover, given that oxidative stress is a central injury mechanism, this raises the question of whether antioxidant supplementation might mitigate some of the harmful effects of SO_2_ in exposed individuals. Finally, the emerging physiological role of endogenous SO_2_ as a gasotransmitter warrants further exploration for its therapeutic and biomarker potential. Meanwhile, policy-related recommendations may include multi-pollutant exposure cohorts with personal monitoring, SO_2_/AAT-targeted interventions, and NAAQS revision prioritizing low-level mixture effects. Altogether, closing the mechanistic gaps and translating laboratory knowledge into evidence-based policies and clinical practices are crucial to reducing the global health burden of SO_2_.

### Limitations of Research

A major constraint in interpreting SO_2_ health data is that SO_2_ is rarely emitted or encountered in isolation outside laboratory settings. In human exposure settings, SO_2_ is almost always present alongside other major air pollutants, including nitrogen oxides, ozone, particulate matter, and additional toxic gases. As a result, effects attributed to SO_2_ in many epidemiological studies may actually reflect the combined impact of multiple pollutants, making it extremely difficult to distinguish the specific contributions of SO_2_. This issue is particularly significant in studies addressing non-respiratory outcomes such as cardiovascular, neurological, or metabolic effects, where the human evidence is notably weak, and causality is especially uncertain.

In addition, restricted access to research published in other languages and inconsistent reporting of SO_2_ emissions and exposures may have narrowed the review’s scope. Ethical constraints further limit controlled human exposure studies to SO_2_, reducing confidence in establishing definitive causal relationships.

Finally, this review is a narrative synthesis rather than a systematic review or meta-analysis. While this approach enables integration of findings across epidemiological, toxicological, and mechanistic studies, it does not allow calculation of precise effect sizes or formal comparisons between studies. Consequently, the interpretations presented should be viewed as qualitative and integrative rather than definitive.

## Data Availability

No new data were created or analyzed in this study. Data sharing is not applicable to this article.

## Supplementary Materials

The following supporting information can be downloaded at: https://www.mdpi.com/article/10.3390/toxics14010100/s1, Table S1: Studies describing respiratory effects of short-term exposure to SO_2_; Table S2: Studies describing respiratory effects of long-term exposure to SO_2_; Table S3: Extrapulmonary effects of SO_2_ exposure.
