# Supplementary material for: Toxic Effects of Sulfur Dioxide: A Review
Source: Toxics. 2026 Jan 21;14(1):100. doi: 10.3390/toxics14010100 (PMC12846270; doi:10.3390/toxics14010100)
Supplement: Supplementary file 1 [file toxics-14-00100-s001.zip › toxics-4075105-supplementary.pdf]

Table S1: Studies describing respiratory effects of short-term exposure to SO_2.

Study	Population / Model	Exposure Concentration	Symptom Onset	Reported Health Effects
Katsouyanni et al. (1997)[39]	Multi-European city time-series analysis	50 µg/m³ increase	Same-day increase	• Partially contributed to a 3% (95% CI[a]: 2%–4%) increase in daily mortality
Samoli et al. (2011)[37]	Athens, Greece	10 µg/m³ increase	Same-day increase	• 5.98% (95% CI: 0.88%–11.33%) increase in pediatric asthma ER[b] visits
Chen et al. (2012)[40]	17 Chinese cities	10 µg/m³ increase in 2-day moving averaged SO_2 levels	Within 1–2 days	• 1.25% (95% PI[c]: 0.78–1.73) increase in SO_2 related respiratory mortality
Li et al. (2021)[41]	Nationwide, multi-city, time-series analysis in China	10 µg/m³ increase in 4-day moving averaged SO_2 levels	Within 1–2 days	• 0.83% rise in YLL[d] from COPD[e] (95% CI: 0.13%–1.53%)<br>• The related mortality increase was 0.78% (95% CI: 0.16%–1.41%)
Orellano et al. (2021)[42]	Systematic review and meta-analysis of 67 included articles	10 µg/m³ increase	Same or next day (lag 0–1)	• Positive correlation between 10 µg/m³ increase in SO_2 levels and all-cause and respiratory mortality
Zhou et al. (2024)[38]	Systematic review and meta-analysis of 15 included articles	10 µg/m³ increase	N/A[f]	• Pooled relative risk (RR[g]) for COPD was 1.26 (95% CI: 0.94–1.70) per 10-µg/m³ increase in ambient SO_2

[a]CI = Confidence interval; used in frequentist approaches for regression or hypothesis testing.

[b]ER = emergency room

[c]PI = More commonly known as credible (posterior) interval; used in Bayesian approaches, especially for small-area estimation, disease mapping, or when prior information is valuable

[d]YLL = Years of Life Lost

[e]COPD = Chronic obstructive pulmonary disease

[f]N/A = Not available or not applicable

[g]RR= Relative risk

Table S2: Studies describing respiratory effects of long-term exposure to SO_2.

**Study**	**Population / Design**	**n**	**Follow-up / Exposure Period**	**Key Respiratory Findings**
Herbarth et al. (2001)[51]	Risk analysis of air pollution and childhood bronchitis in East Germany	3,816 children	~10 yrs	• Bronchitis incidence strongly associated with SO_2 exposure (OR: 3.51, 95% CI: 2.56–4.82)
Lin et al. (2004)[52]	Case-control of childhood asthma hospitalizations, Bronx, New York City	4,865 child hospital admissions (ages 0–14)	June 1991–December 1993 (~2 yrs)	• SO_2 increases were significantly associated with childhood asthma hospitalizations (RR: 1.07, 95% CI: 1.02–1.12 per 24-hr SO_2 increase)<br>• OR: 2.21 peaked at a 3-day lag
Lee et al. (2002)[49]	Multi-national occupational cohort (12 countries; pulp, and paper industry)	57,613 workers (40,704 exposed to SO_2)	1945–1996 (~50 yrs)	• Higher lung cancer risk linked to cumulative SO_2 exposure (RR: 1.49, 95% CI: 1.14–1.96) after adjusting for co-exposures
Andersson et al. (2013)[50]	Occupational cohort of Swedish pulp mill workers with repeated SO_2 gassings	3,060 workers (2,037 in sulfite mills)	1970–2000 (~30 yrs)	• Higher incidence of bronchitis in repeatedly gassed workers (HR[b]: 2.1, 95% CI: 1.4–3.1)<br>• Never-smokers frequently gassed had elevated risk (HR: 8.7, 95% CI: 3.5–22)

Table S3: Extrapulmonary effects of SO_2 exposure.

Source	Organ System / Outcome	Population / Design	Exposure Window	Key Findings
Szyszkowicz et al. (2012)[55]	Neurological - ED visits for stroke & seizure	Vancouver, Canada, case-crossover	1999–2003 (~5 yrs), lag 0-3	• ED visits for stroke (OR: 1.12, 95% CI: 1.02–1.23, lag 3)<br>• ED visits for seizures (OR: 1.18, 95% CI: 1.05–1.32, lag 2, women only)
Liu et al. (2017)[56]	Neurological - stroke hospitalization	14 Chinese cities, multicity case-crossover	2014–2015 (~2 yrs), 6-day average	• Ischemic stroke admissions increased by 1.6% (95% CI: 1.0–2.3%) for every 10.1 μg/m³ increase in ambient SO_2 concentration, corresponding to IQR[a]<br>• No clear effect for hemorrhagic stroke.
Shan et al. (2020)[62]	Metabolic - T2DM[b] incidence & mortality	Northern China, Cohort study	1998–2009 (~12 yrs)	• A 10 μg/m³ increase in SO_2 was associated with increases in both:<br>o Diabetic incidence: (HR: 1.29, 95% CI: 1.26–1.32)<br>o Diabetic mortality: (HR: 1.13, 95% CI: 1.04–1.23)
Wu et al. (2021a)[63]	Metabolic - T2DM mortality	Wuhan, China, time-series	2013–2019 (~7 yrs), lag 0-3	• A 10 μg/m³ increase in SO_2 was associated with a 3.84% increase in daily T2DM deaths (95% CI: 1.48–6.19)<br>• Stronger effects observed in women and adults aged ≥65 years
Wu et al. (2021b)[57]	Cardiovascular - stroke burden	48 Chinese cities, time-series	2013–2017 (~5 yrs), lag 0–3	• A 10 μg/m³ increase in SO_2 was associated with a 0.7% (95% CI: 0.27%–1.13%) increase in YLL from total stroke

Li et al. (2021)[64]	Metabolic - T2DM incidence	Data on 6,426,802 non-diabetic participants aged 30-50 were obtained from the Taiwanese National Health Insurance Research Database Taiwanese adults, time-series	2008–2015 (~7 yrs)	• Each IQR SO_2 increase (1.77 ppb) was associated with HR 1.011 for incident T2DM (95% CI 1.007–1.015)

[a]IQR = Interquartile range

[b]T2DM = type 2 diabetes mellitus
